# *TERT* rs2853676 polymorphisms correlate with glioma prognosis in Chinese population

**DOI:** 10.18632/oncotarget.12064

**Published:** 2016-09-16

**Authors:** Xue He, Yahui Wei, Zhengshuai Chen, Xikai Zhu, Lifeng Ma, Ning Zhang, Yuan Zhang, Longli Kang, Dongya Yuan, Zongyong Zhang, Tianbo Jin

**Affiliations:** ^1^ Key Laboratory for Molecular Genetic Mechanisms and Intervention Research on High Altitude Disease of Tibet Autonomous Region, School of Medicine, Xizang Minzu University, Xianyang, 712082 Shaanxi, China; ^2^ Key Laboratory for Basic Life Science Research of Tibet Autonomous Region, School of Medicine, Xizang Minzu University, Xianyang, 712082 Shaanxi, China; ^3^ Key Laboratory of High Altitude Environment and Gene Related to Disease of Tibet Ministry of Education, School of Medicine, Xizang Minzu University, Xianyang 712082, Shaanxi, China; ^4^ Central Hospital of Xianyang, Xianyang 712000, Shannxi, China; ^5^ National Engineering Research Center for Miniaturized Detection Systems, School of Life Sciences, Northwest University, Xi'an, 710069 Shaanxi, China; ^6^ Life Science Research Centre of Taishan Medical University, Taian, 271016 Shangdong, China

**Keywords:** glioma, TERT, rs2853676, overall survival, progress-free survival

## Abstract

High rates of recurrence and the lack of effective treatments contribute to the poor prognosis of patients with glioma. There is therefore an urgent need for an easily detectable biomarker to facilitate early detection. In this study, we explored the association between *TERT* rs2853676 genetic polymorphisms and the prognosis of Chinese glioma patients. A total of 481 glioma patients at the Tangdu Hospital of the Fourth Military Medical University in China were included in this study. The overall survival rates were calculated using the Kaplan-Meier method. Prognostic factors were determined through multivariate Cox regression analysis. The overall survival (OS) rates of one, two, and three years were 31%, 10.3%, and 7.5%, respectively. The progress-free survival (PFS) rates of one, two, and three years were 15.7%, 7.3%, and 4.7%, respectively. The genotype “A/G” of *TERT* rs2857676 decreased the PFS rate (hazard ratios [HR] = 0.824; *P* = 0.059). The genotype “A/G (HR = 0.803; 95% CI, 0.656 – 0.982; *P* = 0.032)” and “A/A + A/G” decreased the recurrence rate compared to the genotype G/G (HR = 0.818; 95% CI, 0.675-0.99; *P* = 0.040). Our study indicates that *TERT* rs2853676 polymorphisms correlate with glioma survival and recurrence rates in a Chinese population, which suggests that they could potentially serve as prognostic markers in glioma patients.

## INTRODUCTION

Gliomas are the most common and deadly brain tumors of the central nervous system (CNS), which clinically characterized by a high incident rate, a high recurrence rate, and high mortality, accounting for up to 50% of all intracranial tumors [1][2, 3]. Gliomas are classified by World Health Organization (WHO) into four grades in accordance with the morphological resemblance of the neoplastic cells to normal glial tissues [4]. Based on histological criteria, diffuse gliomas are categorized into astrocytoma, oligodendroglioma and oligoastrocytoma, and graded from grade II to IV [5]. Gliomas with diverse genetic variations might originate from distinct cell types and are an important cause of tumor heterogeneity [6, 7]. However, the past two decades have seen the remarkable progress in basic brain tumor biology, especially as far as malignant glioma and medulloblastoma were concerned, the most common CNS cancers of adults and children. For instance, patients with astrocytic glioma, also known as glioblastoma, the most widespread and aggressive glioma variant, have a median survival of only 15 months [8, 9]. Brain tumors are the most common solid tumors affecting children, and the main cause of cancer-related death in children [10]. Despite efforts to improve treatment, children with high-grade glioma still have a dismal outcome with a 5-year survival of less than 20% [11].

Among glioblastoma, patients with grade IV tumors have relatively better, but variable, survivals than patients with grade II and III tumors. Due to their variable prognosis and difficulties in designing and evaluating clinical trials in WHO grade II and III diffuse gliomas, treatment strategies of these gliomas are still controversial [12–14]. The *TERT* gene contains 16 exons and 15 introns spanning about 35 kb, mapped on chromosome 5p15.33 [15]. The maintenance of telomeres requires a number of telomere associated factors. One such complex is the telomerase enzyme, which contains *TERT* and *TERC*, a protein and an RNA component, respectively. *TERT* uses the RNA subunit of telomerase as a template for the synthesis of single stranded DNA in the telomeric region of the chromosome, loading to prevent the chromosome from shorting in nucleotide repeats region. However, because telomerase activity is absent in the process of the most cell differentiation, resulting in telomeres shorten over time. Telomerase are reduced to a certain length, present in most cancer cells, which may block cancer cells from senescence or apoptosis [16, 17].

Because of the poor prognosis of glioma, there is an urgent need for an easily detectable biomarker to facilitate early detection. In this study, we undertook a retrospective analysis of data from a prospective longitudinal study of 481 patients over an extended time period (2010–2014) to examine the epidemiology of glioma with regard to age, gender, surgery, radiotherapy, chemotherapy (platinum, nimustine, and temozolomide), WHO grade, overall survival (OS), and progress-free survival (PFS). We analyzed the correlation between *TERT* rs2853676 genetic polymorphisms and glioma prognosis, to identify possible points of intervention that may lead to improved patient survival.

## RESULTS

From September 2010 to May 2014, a total of 481 glioma cancer patients at the Tangdu Hospital of The Fourth Military Medical University in China were included in this study. Table 1 summarizes the population and clinical characteristics. The average age of all patients (264 men and 217 women) was 40.79 years (ranging from 1 to 81 years); 43.2% of patients were less than 40 years old. 315 (62.5%) patients were treated with gamma knife (GK) radiotherapy, 122 (25.4%) patients were treated with conformal radiotherapy (CRT), and the rest 44 patients did not receive any radiotherapy. 198 (41.2%) patients were treated with chemotherapy, while 283 (58.8%) patients were not treated. There were 294 cases with grade I–II tumors and 187 cases with grade III–IV tumors.

**Table 1:** Patients and clinical characteristics

Variable		No.	Percent (%)
**Gender**	male	264	54.9
	female	217	35.1
**Age**	Mean (range), years	40.79(1-81)	
	<40	208	43.2
	≥40	273	56.8
**Surgery**	GTR	328	68.2
	NTR+STR	153	31.8
**Radiotherapy**	GK	315	62.5
	CRT	122	25.4
	No	44	9.1
**Chemotherapy**	Yes	198	41.2
	No	283	58.8
**WHO Grade**	Grade I-II	294	61.1
	Grade III-IV	187	38.9
**Overall Survival**	Median follow-up	11 months	
	Survival	26	5.4
	Loss to follow-up	17	3.5
	Death	438	91.1
**OS rate, %**	1 year	31	
	2 years	10.3	
	3 years	7.5	
**Progression-free survival**	Median follow-up	8 months	
	Progression-free	24	5
	Progression	453	94.2
	Total	477	99.2
	Missing System	4	0.8
**PFS rate, %**	1 year	15.7	
	2 years	7.3	
	3 years	4.7	
	Total Number	481	

Abbreviations: GTR: gross-total resection; NTR: near-total resection; STR: sub-total resection;

GK: Gamma Knife; CRT: Conformal Radiotherapy; OS: Overall Survival; PFS: Progression-free Survival.

**Figure 1:**
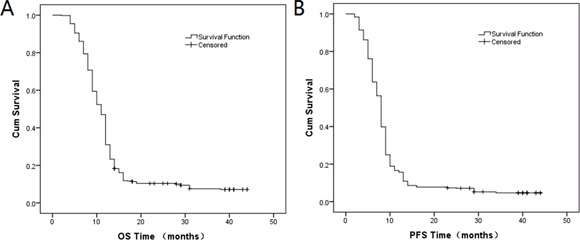
Kaplan-Meier curves of A. OS and B. PFS.

Patients were followed up since the diagnosis until the end of May, 2014. Among the 481 patients, 17 patients were lost from the follow-up data and 438 (91.1%) death cases occurred until the end of the follow-up. The median follow-up time of OS was 11 months, and the OS rates of one year, two years and three years were 31%, 10.3% and 7.5%, respectively. The median follow-up time of PFS was 8 months, and the PFS rates of one year, two years and three years were 15.7%, 7.3%, 4.7%, respectively. OS and PFS curves were drawn using the Kaplan-Meier method in Figure 1A and Figure 1B, respectively.

**Figure 2:**
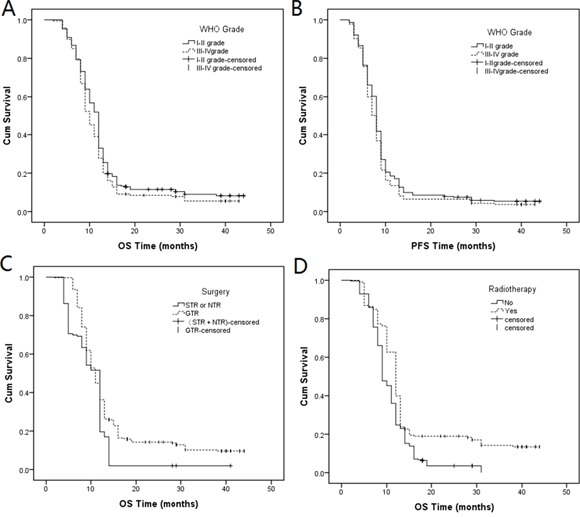
Kaplan-Meier curves of A. OS (log-rank *P* = 0.039) and B. PFS (log-rank *P* = 0.12) between grade I–II and grade III–IV tumors; Kaplan-Meier curves of the distribution of C. surgery (log-rank *P* = 0.000) and D. radiotherapy (log-rank *P* = 0.000) in OS.

As shown in Table 2, we found that there only existed statistical difference in the distribution of age with a *P*-value = 0.001, but for distribution of gender, age, surgery, radiotherapy and chemotherapy between grade I–II and grade III–IV tumors. We also found there was statistical difference in OS (Log-rank *P* = 0.039, Figure 2A) between grade I–II and grade III–IV tumors, but there was no statistical difference in PFS (Log-rank *P* = 0.12, Figure 2B).

**Table 2:** The distribution of variable between grade I–II and grade III–IV tumors

Variable	N/%	N/%	*P*-value^a^
	Grade I-II	Grade III-IV	
**Gender**			0.412
Male	157/53.4	107/57.2	
Female	137/46.6	80/42.8	
**Age**			0.001*
Mean(range)/year	38.63(1-81)	44.81 (2-79)	
<40	144/49	64/34.2	
≥40	150/51	123/65.8	
**Surgery**			0.706
GTR	197/67	131/70.1	
NTR+STR	97/33	56/29.9	
**Radiotherapy**			0.562
GK	197/67	118/63.1	
CRT	73/24.8	4926.2	
No	24/8.2	2010.7	
**Chemotherapy**			0.637
Yes	124/42.2	74/39.6	
No	170/57.8	113/60.4	
**Total**	294	187	
			***P-*value ^b^**
**OS**			0.039*
Median, months	12	10	
Survival	17/5.8	9/4.8	
Loss to follow-up	14/4.8	3/1.6	
Death	263/89.5	175/93.6	
OS rate, % 1 year	33	39	
2 years	11.5	8.5	
3 years	9	5.5	
**PFS**			0.12
Median, months	8	8	
Progression-free	17/5.8	7/3.7	
Progression	275/93.5	178/95.2	
Total	292/99.3	185/98.9	
Missing System	2/0.7	2/1.1	
PFS rate, % 1 year	17.1	13.5	
2 years	7.9	6.5	
3 years	5.4	3.7	

Notes:

a *P*-values based on Pearson χ^2^ test;

b *P*-values based on log-rank test;

* p-value < 0.05 indicates statistical significance.

Abbreviations: GTR: gross-total resection; NTR: near-total resection; STR: sub-total resection; GK: Gamma Knife; CRT: Conformal Radiotherapy; OS: Overall Survival; PFS: Progression-free Survival.

**Figure 3:**
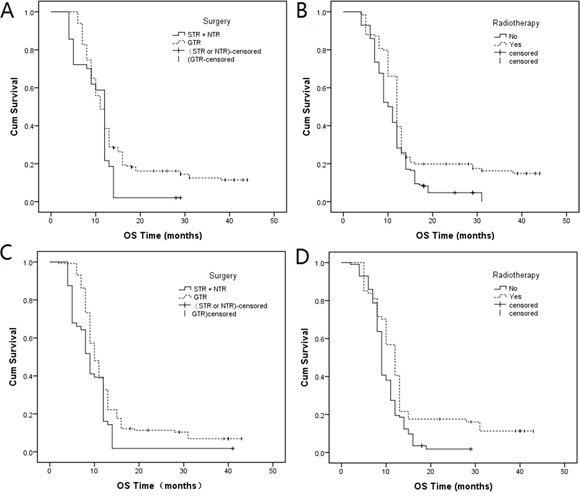
Kaplan-Meier curves of the distribution of A. surgery (log-rank *P* = 0.001) and B. radiotherapy (log-rank *P* = 0.000) in grade I–II OS; Kaplan-Meier curves of the distribution of C. surgery (log-rank *P* = 0.002) and D. radiotherapy (log-rank *P* =0.001) in grade III–IV OS.

In Table 3 indicated that the distribution of surgery and radiotherapy was the following: total OS (Figure 2C, Log-rank *P* = 0.000; Figure 2D, Log-rank *P* = 0.000), grade I–II OS (Figure 3A, Log-rank *P* = 0.001; Figure 3B, Log-rank *P* = 0.000) and grade III–IV OS (Figure 3C, Log-rank *P* = 0.002; Figure 3D, Log-rank *P* = 0.001). Compared with sub-total resection (STR) and near-total resection (NTR) surgery, gross-total resection (GTR) decreased the prognostic risk by 0.667-fold (95% CI: 0.545-0.817, *P* = 0.000), 0.677-fold (95% CI: 0.522-0.878, *P* = 0.003), and 0.617-fold (95% CI: 0.451-0.864, *P* = 0.004) in total OS, grade I–II OS and grade III–IV OS, respectively. Compared with no radiotherapy, radiotherapy treatment decreased the prognostic risk by 0.643-fold (95% CI: 0.528-0.782, *P* = 0.000), 0.665-fold (95% CI: 0.517-0.855, *P* = 0.001), and 0.617-fold (95% CI: 0.450-0.864, *P* = 0.003) in total OS, grade I–II OS and grade III–IV OS, respectively. Genotype “A/G” decreased the prognostic risk by 0.824-fold (*P* = 0.059) in total PFS; other genotypes were not statistically significant, when compared with genotype “G/G”.

**Table 3:** Univariate analysis of patient treatment outcome

				N/Events	Median	*P*-value^a^	1 year	2 years	3 years	HR (95% CI)	*P*-value^b^
**Total**	**OS**	Surgery	STR + NTR	153/150	12	0.000*	19.6	2	2	1	
			GTR	328/288	11		36.3	14.2	10.2	0.667(0.545-0.817)	0.000*
		Radiotherapy	No	283/270	9	0.000*	24.7	3.5	3.5	1	
			Yes	198/168	12		39.9	18.9	14.2	0.643(0.528-0.782)	0.000*
		Genotype	G/G	301/276	11	0.207	28.2	9	6.8	1	
			A/G	150/146	12		37.2	11.8	9.8	0.847(0691-1.038)	0.11
			A/A	24/22	10		25	16.7	0	0.998(0.647-1.542)	0.994
			G/G	301/276	11	0.108	28.2	9	6.8	1	
			A/A + A/G	180/162	11		35.6	12.5	8.8	0.865(0.712-1.050)	0.143
**Total**	**PFS**	Genotype	G/G	299/286	8	0.107	13	5.7	4.1	1	
			A/G	154/143	8		20.8	10.4	6.5	0.824(0.674-1.088)	0.059
			A/A	24/24	8		16.7	8.3	0	0.944(0.622-1.431)	0.785
			G/G	299/286	8	0.044*	13	5.7	4.1	1	
			A/A + A/G	178/167	8		20.2	10.1	5.6	0.839(0.693-1.016)	0.073
**I-II grade**	**OS**	Surgery	STR + NTR	97/95	12	0.001*	21.6	14	14	1	
			GTR	197/168	11		39.6	16.1	12.5	0.677(0.522-0.878)	0.003*
		Radiotherapy	No	170/160	10	0.000*	28.2	4.7	0	1	
			Yes	124/103	12		39.5	19.8	16.3	0.665(0.517-0.855)	0.001*
**III-IV grade**	**OS**	Surgery	STR + NTR	56/55	9	0.002*	16.1	1.8	1.8	1	
			GTR	131/120	10		32.8	11.3	6.9	0.624(0.451-0.864)	0.004*
		Radiotherapy	No	113/110	9	0.001*	19.5	1.8	0	1	
			Yes	74/65	12		40.5	17.6	11.3	0.617(0.450-0.864)	0.003*

Notes:

a *P*-values based on the log-rank test;

b *P*-values based on the Wald test;

* p-value < 0.05 indicates statistical significance.

Abbreviations: GTR: gross-total resection; NTR: near-total resection; STR: sub-total resection; OS: Overall Survival; PFS: Progression-free Survival.

We found that both the genotype “A/G” (95% CI, 0.656-0.982; P = 0.032) and “A/A + A/G” (95% CI, 0.675-0.99; *P* = 0.040) decreased HR 0.8-fold compared to genotype G/G in total PFS by using a multivariate analysis. Other genotypes had no effect on the glioma prognosis. These results are shown in Table 4.

**Table 4:** The association between *TERT* rs2853676 polymorphism and prognosis outcome by multivariate analysis

		Genotype	HR	95% CI	*P*-value
**Grade I-II**	OS	G/G	1		
		A/G	0.873	0.673 - 1.134	0.309
		A/A	0.913	0.493 - 1.689	0.772
	PFS	G/G	1		
		A/G	0.848	0.656 - 1.096	0.207
		A/A	0.916	0.519 - 1.617	0.762
	OS	G/G	1		
		A/A + A/G	0.878	0.682 - 1.128	0.309
	PFS	G/G	1		
		A/A + A/G	0.856	0.67 - 1.094	0.215
**Grade III-IV**	OS	G/G	1		
		A/G	0.735	0.528 - 1.024	0.068
		A/A	0.924	0.492 - 1.736	0.807
	PFS	G/G	1		
		A/G	0.716	0.515 - 0.995	0.047
		A/A	0.852	0.456 - 1.595	0.617
	OS	G/G	1		
		A/A + A/G	0.76	0.555 - 1.04	0.087
	PFS	G/G	1		
		A/A + A/G	0.735	0.538 - 1.004	0.053
**Total**	OS	G/G	1		
		A/G	0.817	0.666 - 1.002	0.052
		A/A	0.944	0.611 - 1.458	0.794
	PFS	G/G	1		
		A/G	0.803	0.656 - 0.982	0.032*
		A/A	0.921	0.607 - 1.398	0.699
	OS	G/G	1		
		A/A + A/G	0.832	0.685 - 1.011	0.064
	PFS	G/G	1		
		A/A + A/G	0.818	0.675 - 0.99	0.040*

Notes: *P*-values based on the Wald test;

* p-value < 0.05 indicates statistical significance.

Abbreviations: HR: hazard ratios; CI: confidence intervals; OS: Overall Survival; PFS: Progression-free Survival.

## DISCUSSION

Making a complete surgical removal is very difficult, due to the tumor cells invade deep into the brain itself. Epidemiological studies have identified several genetic polymorphism loci associated with increased cancer risk on chromosome 5p15.33 [18, 19], which contains two key genes, *CLPTM1L* (*cleft lip and palate transmembrane 1-like*) and *TERT* (*telomerase reverse transcriptase*). In previous genome-wide association studies, researchers have shown that genetic polymorphisms in the telomere-related genes *TERC, TERT* and *RTEL1* are related to increased glioma susceptibility [20–22], suggesting that telomere may play an important role in glioma genesis [23].

*TERT* is the main catalytic subunit of telomerase, and it is essential to maintain of the telomere DNA length [24]. Telomerase is an RNA-dependent DNA polymerase that enrich TTAGGG repetitive sequences, which bind abundant specialized proteins onto the chromosome ends [25]. The telomeres not only prevent coding sequence erosion but also protect chromosomes from rearrangements, fusion and genome instability by conducting chromosomal complete replication and regulating gene expression [26]. It is a vital step for activing the telomerase in the process of cellular immortalization and the malignant transformation of human cells. This activation requires the *TERT* catalyst [27]. One meta-analysis indicated compelling evidence that rs2853676 increased the risk of CNS [28] and another meta-analysis suggested that rs2853676 polymorphism was associated with glioma susceptibility based on 76108 cases and 134215 controls [29]. Given that 60% of tumors being *TERT* promoter mutation, *TERT* is the most frequently mutated gene in gliomas identified thus far [30, 31]. *TERT* promoter mutation glioma patients are typically older [33–35]. In this study, we investigated the relationship between the *TERT* intron mutation and prognosis.

Based on the pathological features, gliomas can be classified as WHO grade I-IV. Almost all high-grade (WHO grade III-IV) gliomas recur after tumor resection. The median survivals of high grade glioma patients are 30-39 weeks [32]. In our study, the median survivals were 40 weeks; this is consistent with previous reports. The median survivals are 5-15 years for patients with WHO grade II glioma [33], while patients suffering from WHO grade III glioma have a median survival of 2-3 years [34, 35]. The antitumor treatment was performed by surgery, radiotherapy, and chemotherapy in combinations. Compared with high-grade patient, low-grade glioma patients have preferable survival, but all low-grade gliomas eventually progress to high-grade gliomas and death. The high rates of recurrence and inefficient treatments contribute to the poor prognosis of patients with gliomas. Especially, high grade gliomas are associated with poor survival, therefore, it is a urgent need for early adjuvant therapies and meticulous follow-up. Despite the development of multi-mode treatments that include surgery, radiotherapy, chemotherapy as well as the emergence of new biological therapies [3, 36], treatment efficacy is still unsatisfactory. In addition, drugs for symptom management are often prescribed for a prolonged period of time, such as corticosteroids and anticonvulsants [37, 38].

Gliomas are usually resistant to therapy even after external beam radiation therapy, aggressive surgical resection and the maximum tolerated chemotherapy dose with agents such as temozolomide or nitrosourea. One of the major strategies is enhancement of chemosensitivity, which can overcome the multidrug and undesirable influence of chemotherapy. The mechanism of chemoresistance is very complicated in tumor therapy and so far, it remains poorly understood [39].

In conclusion, our study indicates that *TERT* rs2853676 polymorphisms correlate with glioma survival and recurrence rates, suggesting that they may serve as diagnostic markers to improve glioma treatment. Further investigations using larger patient numbers and unabridged follow-up data should be carried out to validate this association. Although different symptoms sometimes require different treatment approaches, we recommend a comprehensive treatment approach, which contains pharmacological treatment and/or psychotherapy. To improve treatment efficiency, a better understanding of the molecular mechanisms responsible for glioma recurrence and survival is urgently needed.

## MATERIALS AND METHODS

### Human subjects

All human subjects were informed of the purpose and the experimental procedures of the study. The Human Research Committee of the Tangdu Hospital for Approval of Research Involving Human Subjects approved the use of human tissues in this study. We also obtained signed informed consent from each study participant.

From September 2010 to May 2014, a total of 481 glioma cancer patients (264 men and 217 women) were randomly enrolled at the Tangdu Hospital of the Fourth Military Medical University in Xi'an, China. Their ages ranged from 1 to 81 years, with an average age of 40.79. Patients’ blood samples were collected. Patients underwent a standard clinical examination and none of them had received any therapy before admission before surgery. The histologic diagnosis of tumors was made and agreed upon by at least two senior pathologists at the Department of Pathology of the Hospital. All cases were systematically classified by clinical stage and histological type according to the 2007 WHO classification of tumors of the CNS criteria. The medical records of the patients were reviewed to assess the patients’ characteristics, including age, gender, surgery, radiotherapy, chemotherapy, WHO grade, OS, PFS and final status on the last follow-up examination.

Clinical and follow-up data were collected from medical charts, central radiological systems of the hospitals, out-patient clinics and telephone interviews. PFS was defined as the time from the date of pathological diagnosis to the date of initial tumor recurrence or progression (radiologically or pathologically). OS was measured from the date of pathological diagnosis (September 2010) to the date of death or last follow-up visit (May 2014). The date of death was determined by cancellation of social ID. The survival time was defined as the date from surgery to the date of death. Cases of death patients were regarded as censored data and marked on the survival curves.

### SNP selection and genotyping

The rs2853676 polymorphism has been mapped to intron 2 of the *TERT* gene, which was implicated in the increased risk of glioma in 2009 [21]. We extracted genomic DNA from peripheral blood samples using a GoldMag-Mini Whole Blood Genomic DNA Purification Kit (GoldMag Ltd. Xi'an, China) according to the manufacturer's protocol. Sequenom MassARRAY Assay Design 3.0 Software was used to design primers for amplification and extension reactions [40]. SNP genotyping was performed by Sequenom MassARRAY RS1000 using the standard protocol recommended by the manufacturer Sequenom Typer 4.0 Software was used for data management and analysis [40, 41].

### Statistical analysis

In univariate analysis, differences in PFS and OS between subgroups of patients were evaluated performed by log-rank tests. Survival curves were drawn using the Kaplan-Meier method. A cox proportional hazards model was applied to estimate the risk by calculating hazard ratios (HR) and the 95% confidence intervals (CI) for categorical variables of exposure. Multivariate Cox regression analysis models were then developed that adjusted for the most important covariates, including age, gender, surgery, radiotherapy, WHO CNS classification, chemotherapy, and WHO grade. A multivariate analysis was used taking into account the variables that were found to be significant on univariate analysis. The SPSS statistical software package version 17.0 (SPSS Inc. Chicago, IL, USA) was used for all analyses. *P*-value less than 0.05 were considered statistically significant.

## Abbreviations

OS: overall survival
PFS: progress-free survival
WHO: World Health Organization
CNS: central nervous system
HR: hazard ratios
CI: confidence intervals
GTR: gross-total resection
NTR: near-total resection
STR: sub-total resection
GK: Gamma Knife
CRT: Conformal Radiotherapy
